# Detection of Calpain-Mediated Beclin-1 Cleavage for Drug Discovery in Inflammatory Bowel Diseases

**DOI:** 10.3390/cells15100917

**Published:** 2026-05-18

**Authors:** Kylee A. Hunter, Anne-Marie C. Overstreet, Bryon Benjamin Koff, Hridai Dharan, Steven Overend, Jeannette S. Messer

**Affiliations:** 1 Department of Microbial Sciences in Health, Cleveland Clinic Research, Cleveland Clinic, Cleveland, OH 44195, USAoversta@ccf.org (A.-M.C.O.); koffb@ccf.org (B.B.K.); hsd31@case.edu (H.D.); overens@ccf.org (S.O.); 2 Department of Genetics and Genome Sciences, School of Medicine, Case Western Reserve University, Cleveland, OH 44106, USA; 3 Cleveland Clinic Lerner College of Medicine, Case Western Reserve University, Cleveland, OH 44195, USA; 4 Cleveland Clinic Center for Microbiome and Human Health, Cleveland Clinic Research, Cleveland Clinic, Cleveland, OH 44195, USA

**Keywords:** calpain, inflammatory bowel disease, intestinal epithelial cell, barrier healing, autophagy, bacteria, programmed cell death

## Abstract

**Highlights:**

**What are the main findings?**

- Live commensal bacteria trigger Beclin-1 cleavage, autophagy failure, and programmed cell death mechanisms in cultured intestinal epithelial cells.
- A Beclin-1 cleavage reporter detects the switch between cell survival and cell death states in intestinal epithelial cells challenged by live commensal bacteria.

**What are the implications of the main findings?**

- The Beclin-1 cleavage reporter and associated model of live bacterial stress developed in this study can be used in high-throughput screening assays to identify drug candidates that decrease intestinal epithelial cell death during microbial stress.
- Drugs that decrease intestinal epithelial cell death during microbial stress could restore intestinal barrier integrity, a therapeutic goal in inflammatory bowel diseases.

**Abstract:**

Inflammatory bowel diseases (IBDs) are diseases of chronic inflammation and intestinal epithelial cell (IEC) death that affect an estimated 7 million people worldwide. Intestinal barrier restoration is the most important determinant of remission in IBD, yet there are very few existing therapies that protect IECs from damage or support epithelial repair. The goal of this study was to develop a model system and tools that can be used to identify therapeutics that promote IEC survival in IBD. We developed a Beclin-1 cleavage reporter (BICR) that detects calpain-mediated Beclin-1 cleavage and the switch from autophagy to programmed cell death. We modified BICR with the HIV Tat peptide (BICR-Tat) and tested it in a model of live bacterial stress using commensal *E. coli* and IEC. BICR sensitively and specifically detected calpain activity in cell-free assays, and BICR-Tat successfully detected Beclin-1 cleavage and autophagy failure in IEC. Achieving IEC survival in the microbe-challenged IBD gut would be an important advance toward intestinal barrier restoration in this intractable disease. The BICR-Tat reporter coupled with the model of microbial stress developed in this study could enable high-throughput screening approaches to identify therapeutics with the potential to achieve barrier healing and sustained remission in IBD.

## 1. Introduction

Inflammatory bowel diseases (IBDs) are chronic, often devastating diseases of epithelial damage, inflammation, and immune activation that affect an estimated 7 million people worldwide [1,2]. Epithelial integrity is a key factor in IBD since intestinal barrier healing is the single greatest predictor of disease progression in both types of IBD, Crohn’s disease and ulcerative colitis [3,4]. The intestinal barrier is composed of a single layer of tightly interconnected epithelial cells that regulate the passage of macromolecules across the barrier and contain microbes and their components within the gut lumen [5]. Microbes damage this barrier by directly injuring intestinal epithelial cells (IECs) and activating inflammatory pathways that lead to programmed cell death (PCD) [6,7]. Tissue-adherent bacteria, inflammation, and IEC death are features of active IBD lesions [7]. This suggests that therapeutics targeting microbe-activated PCD pathways would promote intestinal barrier restoration in IBD. However, we currently lack drugs that mitigate microbe-triggered IEC death and have very few tools amenable to the screening pipelines required to develop new drugs to target this aspect of IBD.

High mobility group box 1 (HMGB1) is a ubiquitously expressed protein that plays several roles in IBD [8]. It has been extensively characterized as an extracellular damage-associated molecular pattern molecule (DAMP) that is increased in circulation during a large number of inflammatory diseases, including IBD [9,10,11,12,13]. Exogenous administration of recombinant HMGB1 is reported to increase inflammation in a variety of murine inflammatory disease models, and strategies to block HMGB1 release or activity have been proposed as therapy for IBD and other inflammatory diseases [14]. In contrast, our group reported a cytoprotective role for intracellular HMGB1 in IBD [15]. In IECs exposed to the bacterial cell wall component muramyl dipeptide (MDP), HMGB1 accumulates in the cell cytosol and protects cells from death due to microbe-induced stress. Cytosolic HMGB1 supports autophagy by protecting the core autophagy proteins Beclin-1 and Atg5 from calpain cleavage. Even more importantly, when HMGB1-deficient IECs are exposed to MDP, the calpain cleavage event switches both Beclin-1 and Atg5 from pro-autophagic to pro-apoptotic proteins. Calpain-mediated Beclin-1 and Atg5 cleavage was also seen in IECs from mice conditionally deficient in IEC HMGB1 in both the dextran sodium sulfate and IL-10^-/-^ models of colitis.

Patients with active IBD lesions have increased HMGB1 mRNA but decreased HMGB1 protein in their IECs [15]. Furthermore, the same pathway of Beclin-1 and Atg5 cleavage and apoptosis seen in HMGB1-deficient IECs and in murine colitis with IEC deficiency of HMGB1 is recapitulated in IBD lesions. HMGB1 deficiency and the switch from autophagy to apoptosis occur in active lesions from the small and large intestines and in patients with both ulcerative colitis and Crohn’s disease. In HMGB1-deficient intestinal organoids, calpain inhibition rescues Beclin-1 and Atg5 from cleavage and prevents IEC death during microbial stress. Likewise, calpain inhibition protects Beclin-1 and Atg5, preserving the intestinal epithelial barrier and ameliorating colitis caused by dextran sodium sulfate in mice lacking HMGB1 in IEC. Together, these data suggest that HMGB1 deficiency is a final common pathway of IBD pathophysiology, and that deficiency leads to IEC death when cells experience microbial stress. They also demonstrate that preventing cleavage of Beclin-1 and Atg5 can protect the intestinal epithelial barrier in microbial stress and inflammation. Therefore, manipulating the autophagy/apoptosis checkpoint to favor IEC survival could be a powerful advance in IBD therapeutics.

Calpain activity determines the outcome of autophagy/apoptosis calculations in microbe-stressed IECs [15]. Calpains are a large group of intracellular calcium-dependent cysteine proteases [16]. Characteristically, this group of proteases performs limited proteolysis that affects substrate protein functions, rather than completely degrading substrates [17]. However, other proteases can act on calpain cleavage fragments as well, leading to complete protein degradation [18]. Members of the calpain family are ubiquitously expressed (i.e., Calpain-1 and Calpain-2) or restricted to specific cell types (i.e., Calpain-9 in gut epithelial cells) [19]. Calpains are activated in the context of increased cellular calcium, and activation can be triggered by microbe-associated molecular patterns (MAMPs), bacterial virulence factors that damage cells, or bacterial invasion through cell membranes into cells [20,21,22,23]. When calpains are activated, HMGB1 regulates both the overall calpain activity and calpain action on substrates crucial for cell survival [15]. Low levels of HMGB1 lead to unchecked calpain activity and irreversible modification of Beclin-1 and Atg5 that commit cells to PCD. The most direct therapeutic approach to preventing Beclin-1 and Atg5 cleavage in HMGB1-deficient IECs is to inhibit calpain activity. However, calpains are required for epithelial repair, so inhibition may increase IEC survival in the short term, but would be counterproductive in the long term [24,25,26]. It is also unclear whether the functions and kinetics of different calpains can be untangled to specifically target only the portions that contribute to disease and spare portions needed for healing. For these reasons, strategies that protect vital calpain substrates, like Beclin-1 and Atg5, are more likely to succeed than those that focus solely on suppressing calpain activity.

Here, we report the development of a sensitive, rapid, and cost-effective technique for detecting Beclin-1 cleavage in cell-free and cell-based assays. We designed an 11-amino acid Beclin-1 cleavage reporter (BICR) that contains the Calpain-1 cleavage site flanked by a fluorescent dye and a dye quencher. Cleavage releases the dye from quenching, and the signal is directly proportional to the amount of reporter cleaved. We tested the BICR in IECs challenged with live bacteria. In this model, infection leads to calpain activation and autophagy failure. The BICR faithfully and sensitively reported calpain activity in cell-free assays, including when activity was diminished by known calpain inhibitors. BICR modified with the addition of the HIV Tat peptide sequence was able to penetrate into cultured mammalian cells, where it reported Beclin-1 cleavage by native cell proteases. Together, our data demonstrate the utility of BICR as a tool to probe intra- and extracellular events that lead to Beclin-1 cleavage and identify therapeutic strategies to regulate the autophagy/apoptosis checkpoint. This tool can be used to explore mechanistic pathways that determine cell fate decisions during microbial stress and in drug discovery for diseases of epithelial damage, such as IBD.

## 2. Materials and Methods

### 2.1. BICR Nanosensor Design

To model the calpain cleavage site in Beclin-1, the full-length structure of human Beclin-1 was generated using AlphaFold Monomer v2.0 and structurally visualized using PyMOL3.1.6.1 [27].

To detect Beclin-1 cleavage by calpains, two peptides were created for this study. (1) Beclin-1 cleavage reporter (BICR): A peptide consisting of a Beclin-1 fragment corresponding to a previously identified calpain cleavage site was flanked by a fluorophore (5-FAM (5-Carboxyfluorescein)) and a proprietary quencher (CPQ2) [15]. BICR sequence: 5FAM-PEG2-QEEETNSGEEP-Lys(CPQ2)-NH2 (CPC Scientific, custom product 896291, Rocklin, CA, USA). (2) BICR-Tat: BICR peptide conjugated to the HIV Tat sequence (amino acids 47-57) at the C-terminus. BICR-Tat sequence: 5FAM-PEG2-QEEETNSGEEP-Lys(CPQ2)-PEG2-YGRKKRRQRRR (CPC Scientific, custom product number 896290).

### 2.2. In Vitro BICR Assays

In vitro BICR assays were all performed using the same assay buffer composed of 50 mM 4-(2-Hydroxyethyl)piperazine-1-ethanesulfonic acid) (HEPES; Research Products International, H75030, Mount Prospect, IL, USA)-Sodium Hydroxide (NaOH) pH 7.5, 10 mM 1,4-dithiolretitrol (DTT; Sigma-Aldrich, D0632, St. Louis, MO, USA), and 5 mM Calcium Chloride (CaCl_2_; Sigma-Aldrich, 223506). Reactions were performed at a final volume of 100 μL in black-walled, black-bottom plates (Corning, 3631, Corning, NY, USA) and fluorescence was measured at 485 nm excitation/535 nm emission every 10 min for 1–2 h at room temperature, with agitation between each reading, on a BioTek Synergy H1 plate reader (Agilent, Santa Clara, CA, USA).

To establish the optimal BICR concentration for in vitro assays, the indicated concentrations of BICR (0, 0.1, 0.5, and 1.0 μM) were diluted in reaction buffer, and then native human active Calpain-1 (Calbiochem, 208713, San Diego, CA, USA) was added to the BICR/buffer mixture at a final concentration of 100 nM.

To determine the range of calpain activity that BICR/BICR-Tat was able to detect, serial dilutions of native active calpain-1, both human and porcine (Calbiochem, 208713 and 208712, respectively), were prepared in reaction buffer, and then BICR or BICR-Tat was added to the reaction at a final concentration of 0.5 μM.

To evaluate the specificity of BICR cleavage, porcine calpain-1, recombinant human caspase-1 (MedChemExpress, HY-P72117, Monmouth Junction, NJ, USA), recombinant human caspase-3 (MedChemExpress, HY-P72874), or recombinant human caspase-8 (BPS Bioscience, 80114, San Diego, CA, USA) (final concentrations 50, 75, 100 nM) was added to reaction buffer containing BICR (0.5 μM final concentration).

To test whether BICR could detect calpain inhibition, calpain inhibitors MG-132 (MedChemExpress, HY-13259), Dazcapistat (MedChemExpress, HY-132850), and Calpeptin (MedChemExpress, HY-100223) were diluted in reaction buffer and mixed with BICR (final concentration 0.5 μM), then porcine Calpain-1 was added to the reaction (final concentration 100 nM).

To determine whether HMGB1 can inhibit BICR cleavage by calpain, Calpain-1 (final concentration 50 nM) and HMGB1 (final concentration 50 nM) were diluted in reaction buffer, and then BICR (final concentration 0.5 μM) was added to the reaction.

### 2.3. Comparison Between BICR and Commercial Calpain Substrate 3 Fluorogenic (CS3F)

The assay buffer for these experiments was composed of 50 mM HEPES-NaOH, pH 7.5; 10 mM DTT; and 5 mM CaCl_2_. Reactions were performed at a final volume of 100 μL in black-walled, black-bottom plates. After reactions were mixed, the plates were protected from light and incubated with agitation for 60 min at room temperature. Fluorescence was measured at 485 nm excitation, 535 nm emission on a BioTek Synergy H1 plate reader and readings were normalized against the buffer alone (set to relative fluorescence units = 0).

To compare the activity of BICR and CS3F across a range of concentrations, BICR and Calpain Substrate III, Fluorogenic (CS3F; Calbiochem, 208771) were diluted in reaction buffer, and then porcine Calpain-1 (final concentration 0.1 μM) was added to the reactions.

To compare the sensitivity of BICR and CS3F for the detection of calpain activity, fixed concentrations of BICR (2 μM) and CS3F (10 μM) were prepared in reaction buffer. Porcine Calpain-1 was also serially diluted as indicated in reaction buffer and added to the BICR- or CS3F-containing mixture.

### 2.4. Tat-FAM Entrance into IEC

To quantify Tat peptide entrance into IEC, wild-type (WT) CaCo2 cells (Cyagen, Santa Clara, CA, USA) were seeded at a density of 1.0 × 10^4^ cells/well on a black-walled, black-bottom, 96-well tissue culture plate (CELLSTAR, 655086, Kremsmünster, Austria) and were grown for approximately 3 days prior to use. Cells were washed with Dulbecco’s PBS (DPBS; Genesee Scientific, 25-508, El Cajon, CA, USA), then incubated with 2.5 μM bisBenzimide H 33258 (Hoechst; Sigma-Aldrich, 14530) and 0 μM to 20 μM Tat-Fluorescein (Tat-FAM; Anaspec, AS-61210, Fremont, CA, USA) for 60 min at 37 °C with 5% CO_2_. Cells were washed with DPBS, and fluorescence was measured at 485 nm excitation, 535 nm emission using a BioTek Synergy H1 plate reader.

To visualize Tat peptide entrance into IEC, WT CaCo2 cells were seeded at a density of 1.0 × 10^5^ cells/well and grown on poly-L-lysine-coated coverslips (Electron Microscopy Sciences, 72292-16, Morgantown, PA, USA) for approximately 3 days prior to use. The cells were washed with DPBS, then incubated with 2.5 μM of bisBenzimide H 33258 (Hoechst) and 15 μM Tat-FAM for 60 min at 37 °C with 5% CO_2_. Cells were washed with DPBS and fixed in 4% paraformaldehyde (PFA; Fisher Scientific, 50-980-487, Pittsburgh, PA, USA) for 15 min at room temperature. Cells were then washed with DPBS and mounted with ProLong Glass Antifade Mountant (Thermo Fisher Scientific, P36980, Waltham, MA, USA). Images were captured on a widefield fluorescent microscope (Keyence BZ-800, Broadview Heights, OH, USA).

### 2.5. Immunoblotting

To evaluate autophagy in our model of live bacterial stress, WT and HMGB1-deficient (ΔHMGB1) Caco2 cells were plated at a density of 3.0 × 10^5^ cells/well in a 6-well tissue culture plate and incubated for 3 to 4 days. An overnight culture of *E. coli* SWW33 (gift from Michael Wannemuehler) grown statically in LB broth (Lennox) (LB; Sigma-Aldrich, L3022) was adjusted to an O.D._600nm_ of 2.0 and pelleted for 5 min at 10,000× *g*. Bacteria were resuspended in Dulbecco’s Modified Eagle Medium without phenol red (DMEM; Cleveland Clinic Cell Services, 99BM500CUST DMEM, Cleveland, OH, USA) with 100 nM bafilomycin A1 (Invivogen, tlrl-baf1, San Diego, CA, USA) or DMSO (buffer control; Sigma-Aldrich, D2650). Caco2 cells were washed with DPBS, and *E. coli* SWW33 were added to the Caco2 monolayers at a multiplicity of infection (MOI) of 1:100 for 4 h at 37 °C with 5% CO_2_.

To evaluate the impact of the Tat peptide on Beclin-1 expression and integrity, WT anΔHMGB1 Caco-2 cells (Cyagen) were plated at a density of 3.0 × 10^5^ cells/well in a 6-well tissue culture plate and incubated for 3 to 4 days. The cells were washed with DPBS and incubated with 15 μM Tat-FAM or 15 μM BICR-Tat for 60 min at 37 °C with 5% CO_2_. Lysates were prepared by incubating cells with 1X Cell Lysis Buffer (Cell Signaling Technology, 9803S, Danvers, MA, USA) supplemented with 1 mM Phenylmethylsulfonyl fluoride (PMSF; Goldbio, P-470-25, St. Louis, MO, USA) and cOmplete EDTA-free protease inhibitor cocktail (Sigma-Aldrich, 1183670001) on ice for 10 min and then scraped with a cell scraper. Lysates were clarified by centrifugation at 14,000× *g* for 15 min. The supernatant was transferred to a new 1.5 mL microcentrifuge tube, and protein concentration was determined by bicinchoninic acid assay (BCA; Thermo Fisher Scientific, 23227).

To evaluate Beclin-1 degradation in our model of live bacterial stress, WT Caco2 and HCT-116 cells in a T-75 flask (Genessee Scientific, 25-209) were transfected with pRecieverM01-Beclin-1 (GeneCopoeia, EX-M0768-M01, Rockville, MD, USA) using Lipofectamine 2000 (Invitrogen, Carlsbad, CA, USA) at a DNA: transfection reagent ratio of 1:2 according to the manufacturer’s instructions. The following day, the transfected cells were passaged and seeded at a density of 6.0 × 10^5^ cells/well in a 6-well tissue culture plate and incubated for approximately 2 days. An overnight static culture of *E. coli* SWW33 grown in LB Broth was adjusted to an O.D._600nm_ of 1.2 and pelleted for 5 min at 10,000× *g*. Bacteria were resuspended in Phenol red-free DMEM with 0.5 μM BICR-Tat. Plates were incubated for a range of time between 3.5 and 4 h at 37 °C with 5% CO_2_. Lysates were prepared by incubating cells with 1X Cell Lysis Buffer supplemented with 1 mM PMSF and cOmplete EDTA-free protease inhibitor cocktail on ice for 10 min and then scraped with a cell scraper. Lysates were clarified by centrifugation at 14,000× *g* for 15 min. The supernatant was transferred to a new 1.5 mL microcentrifuge tube, and protein concentration was determined by bicinchoninic acid assay (BCA; Thermo Scientific, 23227).

Lysates were prepared at a protein concentration of 1.5 μg/μL with 4× NuPAGE LDS sample buffer (Invitrogen) supplemented with 0.5 M DTT. 30 μg of each sample was subjected to SDS-PAGE under reducing conditions on a NuPAGE 4–12% gel (Thermo Fisher Scientific, NP0335BOX) with NuPAGE MOPS buffer (Invitrogen, NP0001), and protein was transferred to Immobilon-FL PVDF membrane (Millipore Sigma, IPFL00010, Burlington, MA, USA) with 0.45 μm pore size on ice at 30 V for 60 min. Membranes were blocked with Intercept (PBS) blocking buffer (LI-COR BIOSciences, 927-70003,Bourne, MA, USA) for 60 min at room temperature with agitation.

All primary antibodies were prepared in 1:1 Intercept (PBS) blocking buffer:PBS-T (137 mM NaCl (Thermo Fisher Scientific, S671), 2.7 mM KCl (Thermo Fisher Scientific, P217), 10 mM Na_2_HPO_4_ (Acros Organics, 204855000, Geel, Belgium), 1.8 mM KH_2_PO_4_ (Thermo Fisher Scientific, P285-3), and 0.1%*_w/v_* Tween-20 (Fisher Scientific, BP337-500)). The membranes were first stained with mouse primary antibodies (1 μg/mL mouse anti-LC3 monoclonal [8E10] (MBL Life Science, M186-3, Aichi, Japan), 0.312 μg/mL mouse anti-β-actin monoclonal [8H10D10] (Cell Signaling Technologies, 3700), 0.282 μg/mL mouse anti-GAPDH monoclonal [D4C6R] (Cell Signaling Technologies, 97166), or 0.2 μg/mL THE His tag mouse monoclonal (GenScript, A01800-100, Piscataway, NJ, USA)) at 4 °C overnight with agitation. After staining with mouse primary antibody, membranes were washed with PBS-T for 5 min at room temperature with agitation three times. Membranes were then stained with rabbit primary antibodies (0.17 μg/mL anti-HMGB1 monoclonal [EPR3507] (Abcam, ab79823, Waltham, MA, USA), 1:1000 anti-p62(SQSTM1) polyclonal (MBL Life Science, PM045), 0.074 μg/mL anti-Beclin-1 polyclonal (Cell Signaling Technologies, 3738), or 0.1 μg/mL rabbit β-actin [HL1926] (GeneTex, GTX637675, Irvine, CA, USA)) at 4 °C overnight with agitation. After staining with rabbit primary antibodies, membranes were washed with PBS-T for 5 min at room temperature with agitation three times.

Protein was visualized by first staining with 0.05 μg/mL IRDye 800CW Donkey anti-mouse secondary antibody (LICORbio, 92-32212, Lincoln, NE, USA) prepared in 1:1 Intercept (PBS) blocking buffer:PBS-T supplemented with 0.01% SDS (Thermo Fisher Scientific, BP166) for 60 min at room temperature. Membranes were washed with PBS-T for 5 min at room temperature with agitation three times, then stained with 0.05 μg/mL IRDye 680LT Donkey anti-rabbit secondary antibody (LICORbio, 926-68023) prepared in 1:1 Intercept (PBS) blocking buffer:PBS-T supplemented with 0.01% SDS for 60 min at room temperature. Membranes were washed with PBS-T for 5 min at room temperature with agitation three times, washed once with PBS, then imaged using the Odyssey CLx imager (LICORbio).

### 2.6. BICR-Tat and Calpain Substrate IV Fluorogenic (CS4F) Cleavage in E. coli Exposed Cells

To quantify BICR-Tat and CS4F cleavage in cells stressed by live microbes, WT andΔHMGB1 CaCo2 cells were seeded at a density of 1.0 × 10^4^ cells/well on a black-walled, black-bottom, 96-well tissue culture plate. An overnight static culture of *E. coli* SWW33 grown in LB broth (Lennox) was adjusted to an O.D._600nm_ of 2.0 and pelleted for 5 min at 10,000× *g*. The pellet was resuspended in Phenol red-free DMEM only, 20 μM BICR-Tat in DMEM, or 15 µM CS4F (Calbiochem, 208773) in DMEM. Caco2 cells were washed with DPBS, then 50 µL of DMEM, BICR-Tat, or CS4F mixed with bacteria was added to each well. Plates were incubated for 24 h at 37 °C with 5% CO_2_ and read at 485 nm excitation, 535 nm emission on a BioTek Synergy H1 plate reader.

To visualize BICR-Tat cleavage in cells stressed by live microbes, WT and ΔHMGB1Caco2 cells were plated at a density of 3.0 × 10^4^ cells/well in an 8-well chambered coverslip (Ibidi, 80806, Fitchburg, WI, USA) and grown for 3–4 days. An overnight static culture of *E. coli* SWW33 grown in LB broth (Lennox) was adjusted to an O.D._600nm_ of 2.0 and pelleted for 5 min at 10,000× *g*. The pellet was resuspended in Phenol red-free DMEM or DMEM with 20 µM BICR-Tat. Caco2 cells were washed with DPBS, and then 200 µL of *E. coli* SWW33 with or without BICR-Tat was added to the chamber slide. Slides were incubated for 24 h at 37 °C with 5% CO_2_. After 24 h incubation with *E. coli* SWW33, the media was removed, and cells were washed with DPBS. Cells were fixed in 4% PFA for 15 min at room temperature, washed with DPBS, and then stained with 10 µg/mL bisbenzimide H 33258 (Hoechst) dissolved in TBS at room temperature for 20 min. Following staining, cells were washed with DPBS. Fresh DPBS was added for storage and to prevent drying. After staining and imaging, slides were stored at 4 °C. Images were taken on a widefield fluorescent microscope (Keyence BZ-800).

To quantify BICR-Tat cleavage in cells treated with known protease inhibitors during infection, ΔHMGB1Caco2 cells were plated at a density of 1.0 × 10^4^ cells/well in a black 96-well tissue culture plate with an optical bottom (Ibidi, 89606). After 3 to 4 days of growth, cells were washed with DPBS, then pretreated with 5 µM calpastatin peptide (Calbiochem, 208902), cOmplete EDTA-free protease inhibitor cocktail containing 1 mM PMSF, 1.5 µM Dazcapistat, or Phenol red-free DMEM for one hour. An overnight static culture of *E. coli* SWW33 grown in LB broth (Lennox) was adjusted to an O.D._600nm_ of 2.0 and pelleted for 5 min at 10,000× *g* and resuspended with the corresponding protease inhibitors in DMEM alone or DMEM with 20 μM BICR-Tat and added to the plate as above. Plates were incubated for 24 h at 37 °C with 5% CO_2_ and read at 485 nm excitation, 535 nm emission on a BioTek Synergy H1 plate reader.

Following quantification of BICR, cells were counted in each well of the plate. Cells were washed with DPBS and fixed in 4% PFA for 15 min at room temperature, washed again with DPBS, and then stained with 10 µg/mL bisbenzimide H 33258 (Hoechst) dissolved in TBS at room temperature for 20 min. Following staining, cells were washed with DPBS, fresh DPBS was added to the wells, and plates were stored at 4 °C. Cells were quantified based on the bisbenzimide H 33258 (Hoechst) signal using a BioTek Lionheart FX Automated Microscope (Agilent) with the Gen5 3.18 software.

To establish optimal BICR-Tat concentration for cellular assays with live microbes, WT Caco2 cells were plated at a 1.0 × 10^4^ cells/well in a black-walled, clear-bottom, 96-well tissue culture plate (Stellar Scientific, LI-TC-BK-7491, Baltimore, MD, USA) for 3 to 4 days. An overnight static culture of *E. coli* SWW33 grown in LB broth (Lennox) was adjusted to an O.D._600nm_ of 2.0 and pelleted for 5 min at 10,000× *g*. The pellet was resuspended in Phenol red-free DMEM only, or in 0.625, 1.25, 2.5, 5, or 10 μM BICR-Tat. The Caco2 cells were washed with DPBS, then 50 μL of DMEM, or BICR-Tat mixed with bacteria was added to each well. Plates were incubated for a total of 24 h at 37 °C with 5% CO_2_. The fluorescence was measured at 0, 0.5, 1, 2, 4, 6, 8, 10, 12, and 24 h at 485 nm excitation, 535 nm emission on a BioTek Synergy H1 plate reader. Following the fluorescence measurement at 24 h, a 10X solution of a Caspase-3/-7 detection agent (Thermo Fisher Scientific, C10430) was prepared per the manufacturer’s protocol, and 5 μL of the 10X solution was added to each well. The cells were incubated with the Caspase-3/-7 detection reagent for 60 min at 37 °C with 5% CO_2_. The fluorescence was measured at 550 nm excitation, 590 nm emission on a BioTek Synergy H1 plate reader.

To establish optimal bacterial MOI for cellular assays with live microbes, WT Caco2 cells were plated at a 1.0 × 10^4^ cells/well in a black-walled, clear-bottom, 96-well tissue culture plate for 3 to 4 days. An overnight static culture of *E. coli* SWW33 grown in LB broth (Lennox) was adjusted to an O.D._600nm_ of 2.0, 1.0, and 0.5 and pelleted for 5 min at 10,000× *g*. The pellet was resuspended in Phenol red-free DMEM only or in DMEM with 0.5 μM BICR-Tat. The Caco2 cells were washed with DPBS, then 50 μL of DMEM, or BICR-Tat mixed with bacteria was added to each well. Plates were incubated for a total of 24 h at 37 °C with 5% CO_2_. The fluorescence was measured at 0, 0.5, 1, 2, 4, 6, 8, 10, 12, and 24 h at 485 nm excitation, 535 nm emission on a BioTek Synergy H1 plate reader.

To evaluate BICR-Tat capability with multiple cell types in our model of live bacterial stress, HT29 and HCT116 cells were plated at a density of 1.0 × 10^4^ cells/well on a black-walled, black-bottom, 96-well tissue culture plate. An overnight static culture of *E. coli* SWW33 grown in LB broth (Lennox) was adjusted to an O.D._600nm_ of 2.0 and pelleted for 5 min at 10,000× *g*. The pellet was resuspended in Phenol red-free DMEM only, or in 0.5 μM BICR-Tat. The Caco2 cells were washed with DPBS, then 50 μL of DMEM, or BICR-Tat mixed with bacteria was added to each well. Plates were incubated for a total of 24 h at 37 °C with 5% CO_2_. The fluorescence was measured at 0, 0.5, 1, 2, 4, 6, 8, 10, 12, and 24 h at 485 nm excitation, 535 nm emission on a BioTek Synergy H1 plate reader.

To evaluate BICR-Tat in an IBD-relevant context, WT Caco2 cells were plated at a density of 1.0 × 10^4^ cells/well in a black-walled, clear-bottom, 96-well tissue culture plate. After 3 to 4 days of growth, cells were washed with DPBS, then pretreated with 25, 15, and 5 µM of JSH-23 (MedChemExpress, HY-13982) or DMEM without phenol red for one hour. An overnight static culture of *E. coli* SWW33 grown in LB broth (Lennox) was adjusted to an O.D._600nm_ of 2.0 and pelleted for 5 min at 10,000× *g* and resuspended with 0.5 µM BICR-Tat and the corresponding concentrations of JSH-23 or DMEM alone and added to the plate. Plates were incubated for 24 h at 37 °C with 5% CO_2_ and read at 485 nm excitation, 535 nm emission on a BioTek Synergy H1 plate reader.

### 2.7. Quantification and Statistics

The Z-factor of the BICR-Tat assay was calculated as [28]:

$$Z-factor=1-\frac{3(\sigma_{+}+\sigma_{-})}{\left|\mu_{+}-\mu_{-}\right|}$$

where $\sigma_{+}$ and $\sigma_{-}$ denote the standard deviation of the SWW33 treated samples and samples without bacteria, respectively. Similarly, $\mu_{+}$ and $\mu_{-}$ denote the mean of the *E. coli* SWW33-treated samples and samples without bacteria, respectively.

All data are reported as mean ± s.d. and each datapoint represents one individual biological replicate, unless otherwise noted. In all figures, scale bars = 100 µm, and original magnification is 400X, unless otherwise noted. Significance of differences determined by one-way or two-way ANOVA with a Tukey post hoc test for multiple comparisons. Each experiment is representative of 3 independent replicates (cells, bacteria, and reagents prepared independently). More detailed information about statistical comparisons is noted in the figure legends.

## 3. Results

### 3.1. Development of a Nanosensor to Detect Calpain-Dependent Cleavage of Beclin-1

**Figure 1:**
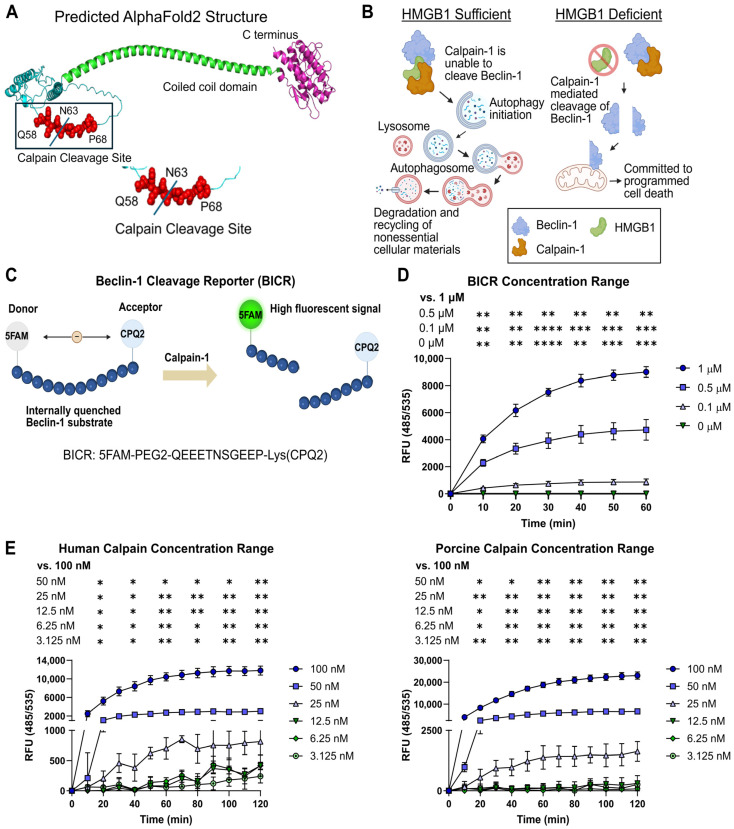
Development of a nanosensor to detect calpain-dependent cleavage of Beclin-1. (**A**), Predicted structure of human Beclin-1 with previously identified calpain cleavage site highlighted. (**B**), Model of how HMGB1 regulates calpain cleavage of Beclin-1. (**C**), Model of the BICR construct. (**D**), Fluorescent signal from BICR cleavage by human Calpain-1 across a range of BICR concentrations. (**E**), Fluorescent signal from BICR cleavage by a range of human and porcine Calpain-1 concentrations. Results reported as the mean ± SD and representative of 3 independent experiments with *n* = 3. * *p* < 0.05, ** *p*< 0.01, *** *p* < 0.001 and **** *p* < 0.0001 according to a two-way ANOVA.

We previously reported that HMGB1 regulates calpain cleavage of Beclin-1 at position N63 and that regulation is disrupted in IBD, leading to activation of the pro-apoptotic function of Beclin-1 (Figure 1A,B) [15]. Here, we used a short peptide derived from Beclin-1 (amino acids 58-68) that contains the calpain cleavage site to create a reporter construct. The peptide, Beclin-1 cleavage reporter (BICR), was flanked by 5-Carboxyfluorescein (FAM), a fluorescent dye, and CPQ2, a quencher that prevents FAM from fluorescing when the two molecules are in close proximity (within approximately 10–100 angstroms) (Figure 1C). Cleavage releases FAM from quenching, resulting in a fluorescent signal that is proportional to the amount of reporter cleaved. The same design principle has been used for other protease reporters [29]. In most cases, these reporters are designed around the optimal substrate for a given protease. Instead, BICR is designed to report cleavage of the substrate of interest, Beclin-1. Since the sequence and cleavage conditions are similar between Beclin-1 and Atg5, detection of BICR cleavage also implies Atg5 cleavage in a given cell.

We first tested BICR performance in vitro using purified Calpain-1. To determine the range of detection, we tested a series of BICR and Calpain-1 concentrations (Figure 1D,E). Results indicated that a 0.5 µM concentration of BICR could detect Calpain-1 concentrations as low as 50 nM. The 0.5 µM concentration of BICR was chosen because it had the highest fold change in fluorescence between baseline and maximal cleavage in the assay. We utilized Calpain-1 of both human and porcine origin in these assays, and the results were similar. Human Calpain-1 is most biologically relevant for the development of new IBD therapies, but porcine Calpain-1 is more cost-effective, which is important for our goal of adapting BICR to high-throughput screening (HTS) pipelines.

### 3.2. BICR Detects Calpain-1 Activity In Vitro

**Figure 2:**
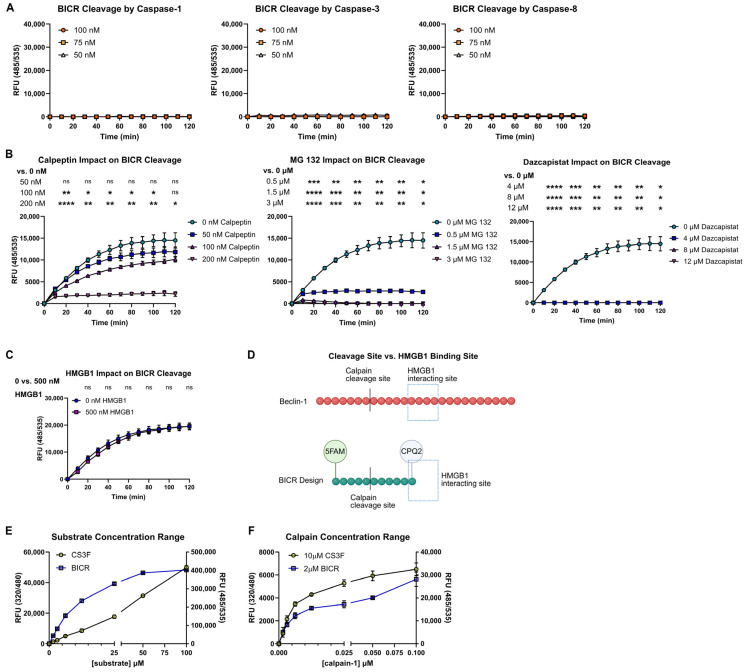
BICR detects Calpain-1 activity in vitro. (**A**), Fluorescent signal from BICR cleavage by Caspase-1, Caspase-3, or Caspase-8. (**B**), Fluorescent signal from BICR cleavage in the presence of the indicated calpain inhibitors. (**C**), Fluorescent signal from BICR cleavage by Calpain-1 in the presence of HMGB1. (**D**), Model of the BICR construct versus Beclin-1 with the HMGB1 interaction site highlighted. (**E**,**F**), Fluorescent signal from CS3F and BICR over a range of substrate (**E**) or porcine Calpain-1 (**F**) concentrations. Results reported as the mean ± SD and representative of 3 independent experiments with *n* = 3. ns = *p* > 0.05, * *p* < 0.05, ** *p* < 0.01, *** *p* < 0.001 and **** *p* < 0.0001 according to two-way ANOVA.

Our goal was to develop a reporter that specifically detects Beclin-1 cleavage by calpains, so it was important to rule out potential off-target activity. In our previous study, Caspase-1 activity accompanied calpain activation in HGMB1-deficient (ΔHMGB1) IECs. A bioinformatic search for protease recognition sites within BICR suggested that it is not a substrate for intracellular proteases aside from calpains [30]. As expected, when BICR was incubated with Caspase-1, Caspase-3, or Caspase-8, there was no evidence of cleavage (Figure 2A).

We next determined whether BICR could identify direct calpain inhibitors in vitro. The addition of known calpain inhibitors dose-dependently decreased BICR fluorescence generated by Calpain-1 (Figure 2B). We also verified that HMGB1 did not influence BICR cleavage (Figure 2C). This was as expected since the HMGB1 binding site in Beclin-1 is absent from BICR (Figure 2D). Thus, BICR can be used to detect calpain activity or its inhibition in vitro. The nature of the BICR construct puts it in the category of a nanosensor, and our results demonstrate that it sensitively and specifically detects calpain cleavage of a peptide derived from Beclin-1, a substrate vital for IEC survival during microbial stress.

Commercial calpain activity assays and reporter substrates are widely available. Generally, these substrates are optimized to detect activity of ubiquitous calpains, such as Calpain-1. Our in vitro assays demonstrated that BICR was efficiently cleaved by Calpain-1, so we tested our cleavage reporter against a commercial substrate (CS3F) to compare the ability of the two reporters to detect calpain activity. The increase in fluorescence generated by BICR or commercial substrate cleavage was comparable over a range of substrate and Calpain-1 concentrations, although the fluorescence intensity of the BICR sensor was consistently higher than that of the commercial reporter (Figure 2E,F). This is likely due to the fact that the two reporters carry different fluorophore-quencher pairs. The kinetics of cleavage were also different between the two reporters, with maximum fluorescence from the commercial reporter after only 15 min, whereas maximum fluorescence from BICR occurred at 1 h.

### 3.3. Tat Conjugation Does Not Affect BICR Activity and Allows Constructs to Enter IECs

The calpains are an important family of proteases that have physiologic functions across virtually every cell type in the human body. Calpains are also of great interest in medicine since they have been implicated in the pathophysiology of IBD, genetic disorders related to gain or loss of calpain function, neuromuscular diseases such as Alzheimer’s disease, metabolic diseases such as Type II diabetes, respiratory diseases such as asthma, and cancers of many different types [15,31,32,33,34]. This has led to a search for methods to characterize and manipulate calpain activity for clinical applications. Most strategies for characterizing calpain activity are centered on detecting calpain activity and inferring effects on substrates. However, changes in substrate activity appear to be most important in human disease. Most of the commercially available fluorogenic calpain cleavage reporters are optimized for calpain cleavage and are non-specific as to substrate. They are also most commonly employed in low-throughput assays where cells can be lysed prior to the assay or for in vitro screens that identify direct calpain inhibitors. To overcome these limitations, several investigators have developed fluorescence resonance transfer (FRET)-based reporters that can be expressed in cells either from plasmids or germline incorporation [35,36,37]. These are powerful tools, but require modification of each cell type of interest and each substrate protein of interest. Another approach has been the use of cell-penetrating peptides to deliver substrate reporters into cells. Among the most interesting and thoroughly tested of these cell-penetrating peptides is the Tat peptide derived from HIV [38].

**Figure 3:**
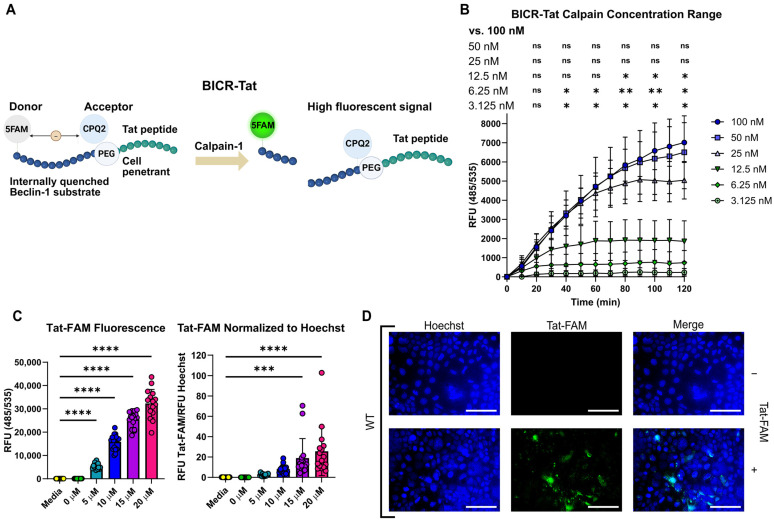
Tat conjugation does not affect BICR activity and allows for constructs to enter IECs. (**A**), Model of the BICR-Tat construct. (**B**), Fluorescent signal from BICR-Tat cleavage by Calpain-1. (**C**), Fluorescence of Tat-FAM in WT Caco2 cells. Raw fluorescence and fluorescence normalized to cell number (Hoechst) are reported. (**D**), Fluorescent signal of Tat-FAM (green) in WT Caco2 cells. Results reported as the mean ± SD and representative of 3 independent experiments performed with *n* = 3 (**B**,**D**) or *n* = 16 (**C**). ns = *p* > 0.05, * *p* < 0.05, ** *p* < 0.01, *** *p* < 0.001 and **** *p* < 0.0001 according to a two-way (**B**) or one-way (**C**) ANOVA. Scale bars—100 µm, and original magnification is 400X.

Our initial studies indicated that BICR performed well in vitro, so we explored Tat-conjugation for the use of BICR in cellular assays. We appended Tat to the C-terminus of BICR to enable live cell monitoring of Beclin-1 cleavage in cultured cells (Figure 3A). While the BICR-Tat contains the same Beclin-1-derived peptide flanked by FAM and CPQ2 as BICR, the addition of the Tat sequence had the potential to impact the ability of calpains to cleave the sequence. To test whether Tat affected BICR cleavage, we assayed BICR-Tat cleavage by Calpain-1 in vitro. Similar to the original BICR, there was a dose-dependent increase in fluorescence after incubation with Calpain-1 (Figure 3B). Interestingly, the signal was lower than the original BICR, but the fold change over baseline was greater.

To test whether Tat could facilitate BICR uptake by IEC, we utilized Tat conjugated to the fluorescent dye incorporated into BICR and FAM. The FAM in Tat-FAM is unquenched, so unlike BICR, it can be observed without any modification of the construct (i.e., cleavage). We observed a dose-dependent increase in fluorescence when Tat-FAM was incubated with wild-type (WT) Caco2 cells, an IEC cell line (Figure 3C). Fluorescent microscopy verified that the FAM signal was intracellular (Figure 3D). Therefore, Tat-conjugation is a feasible method for the delivery of BICR to the IEC cytosol.

### 3.4. Exposure to Live Bacteria Leads to Beclin-1 Cleavage and Autophagy Failure in IECs

In a previous study, we demonstrated that ΔHMGB1 cells exposed to an intracellular MAMP (muramyl dipeptide) experience high and sustained calpain activity that cleaves Beclin-1 and leads to IEC death [15]. Intestinal epithelial cells within active IBD lesions are deficient in intracellular HMGB1 and have evidence of Beclin-1 cleavage as well as activation of PCD. Inhibiting calpain activity increased survival of ΔHMGB1 IECs in both cellular and pre-clinical models of IEC stress. These factors suggest that preventing Beclin-1 cleavage in HMGB1-deficient cells could be a novel strategy for preserving epithelium and/or promoting intestinal barrier healing in IBD.

**Figure 4:**
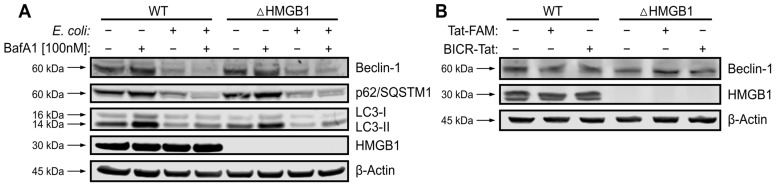
Exposure to live bacteria leads to Beclin-1 cleavage and autophagy failure in IEC. (**A**), Immunoblotting (IB) for Beclin-1, p62/SQSTM1, LC3B, and HMGB1 in Caco2 cells at 4 hpi. (**B**), IB for Beclin-1 and HMGB1 in Caco2 cells exposed to Tat-FAM. Representative of 3 independent experiments.

In the normal gut, IECs are constantly exposed to MAMPs and microbial products generated by commensal bacteria [39]. In IBD, the acellular barrier between bacteria in the gut lumen and IECs fails, allowing for contact between host tissues and gut bacteria [40]. This led us to model stress induced by live bacteria in ΔHMGB1 IECs. We utilized a well-characterized strain of commensal *E. coli* (SWW33) and a human IEC cell line (Caco2, BBe subline) in order to evaluate BICR performance in a format amenable to high-throughput screening [41]. We first investigated Beclin-1 cleavage and the balance between autophagy and apoptosis in this model. Beclin-1 depletion was seen only in infected cells and was associated with a decrease in lipidated LC3B (LC3-II) and in p62/SQSTM1, which is consistent with autophagy failure (Figure 4A). With bacteria, there was evidence of autophagy failure in both WT and ΔHMGB1 cells. The autophagy adaptor p62 generally increases when autophagy fails in healthy cells, but can be cleaved by cellular proteases involved in inflammation and PCD mechanisms [42,43]. We also verified that exposure to the Tat peptide does not alter Beclin-1 expression or trigger Beclin-1 cleavage in these cells (Figure 4B). Thus, the calpain-mediated switch from autophagy to apoptosis is activated in this model of live bacterial stress.

### 3.5. BICR-Tat Reports Beclin-1 Cleavage in IECs Exposed to Live Bacteria

**Figure 5:**
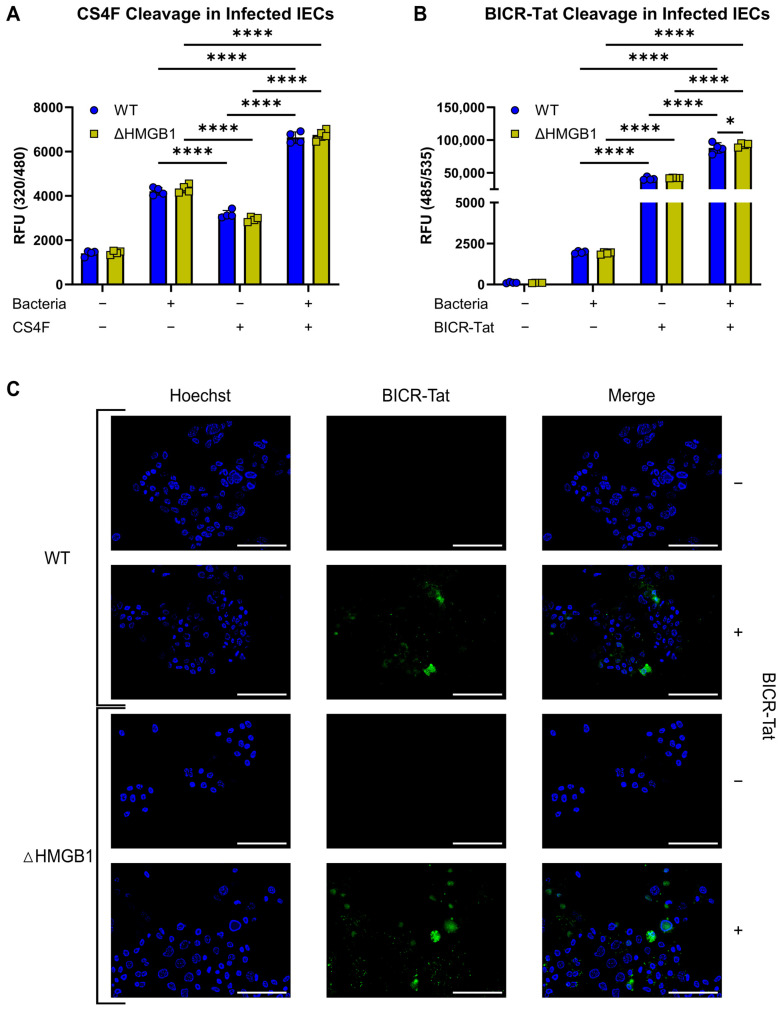
BICR-Tat reports Beclin-1 cleavage in IECs exposed to live bacteria. (**A**), Fluorescent signal from CS4F cleavage in infected WT and ΔHMGB1 cells at 24 hpi. (**B**), Fluorescent signal from BICR-Tat cleavage in infected WT and ΔHMGB1 cells at 24 hpi. (**C**), Fluorescent signal from BICR-Tat cleavage (green) in infected WT and ΔHMGB1 cells at 24 hpi. Results reported as the mean ± SD and representative of 3 independent experiments performed with *n* = 4. * *p* < 0.05 and **** *p* < 0.0001 according to a two-way ANOVA. Scale bars—100 µm, and original magnification is 400X.

We next evaluated calpain activity in our model using a commercially available calpain substrate (CS4F) that was coupled to a Tat sequence for entrance into cells. We detected a significant increase in calpain activity in bacteria-challenged WT and ΔHMGB1 cells at 24 hpi (Figure 5A). This was a later timepoint than we evaluated in our earlier studies because pilot studies with the commercial calpain substrate indicated that significant differences in calpain activity were delayed in comparison to assays run in the absence of calpain substrate. This was not surprising since reporter constructs often dampen or delay protease effects in cells by consuming active protease. We attempted to visualize the calpain reporter by fluorescence microscopy and were unsuccessful. Thus, exposure to live commensal bacteria activates calpains in IECs.

Finally, we assessed Calpain-activated Beclin-1 cleavage in this model using our BICR-Tat reporter construct. There was a significant increase in BICR-Tat cleavage at 24 hpi, in both WT and ΔHMGB1 cells (Figure 5B). The increase in BICR-Tat signal correlated with increased cleavage of the Tat-conjugated commercial calpain substrate at the same timepoint. Microscopic examination of bacteria-challenged cells demonstrated BICR-tat cleavage with higher levels in ΔHMGB1 cells (Figure 5C). Cells were examined before and after fixation, and the appearance of the BICR-Tat signal was similar in both conditions. Interestingly, there appeared to be fewer ΔHMGB1 cells in the BICR-Tat-negative condition than in the BICR-Tat-positive condition. This is again consistent with calpain consumption and protection from PCD by the BICR-Tat peptide itself. This series of experiments demonstrated calpain activation and BICR-Tat cleavage in our model, with a higher level of BICR-Tat cleavage in ΔHMGB1 cells.

### 3.6. BICR-Tat Can Be Used to Identify Compounds That Shift Cells Toward Survival at the Autophagy/Apoptosis Checkpoint During Microbial Stress

We previously discovered that calpain inhibition increased the survival of ΔHMGB1 IECs during MAMP stress [15]. Here, we tested whether calpain inhibition could also rescue ΔHMGB1 IECs during stress caused by live bacteria. We considered this proof-of-concept testing for the use of BICR-Tat in high-throughput screening assays. Since our goal was to mimic IECs in active IBD lesions, we utilized ΔHMGB1 cells in these assays. We also switched from black-walled, black-bottom tissue culture plates to black-walled, optical-bottom tissue culture plates.

**Figure 6:**
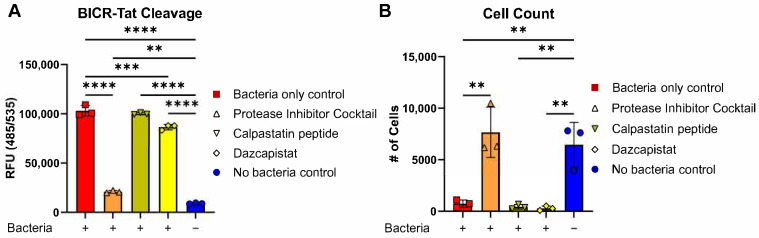
BICR-Tat can be used to identify compounds that increase IEC survival by regulating the autophagy/apoptosis checkpoint during microbial stress. (**A**), Fluorescent signal from BICR-Tat cleavage in infected ΔHMGB1 cells treated with the indicated protease inhibitors, assayed at 24 hpi. (**B**), Cell numbers in samples evaluated in (**A**). Results reported as the mean ± SD and representative of 3 independent experiments with *n* = 4. ** *p* < 0.01, *** *p* < 0.001 and **** *p* < 0.0001 according to a two-way ANOVA.

To test the ability of our assay to identify agents that promote survival decisions at the autophagy/apoptosis checkpoint, ΔHMGB1 cells were treated with known protease inhibitors and challenged with live bacteria. We found that broad-spectrum protease inhibition or the calpain inhibitor dazcapistat decreased BICR-Tat cleavage (Figure 6A). Interestingly, the switch in plate type alone vastly improved the signal-to-noise characteristics of the BICR-Tat cleavage assay on the plate reader. High levels of BICR-Tat cleavage were also associated with high levels of cell death (Figure 6B). Together, these data demonstrate the utility of BICR-Tat for the identification of therapeutics with the potential to limit epithelial damage and promote barrier healing in the damaged gut.

### 3.7. BICR-Tat Cellular Assay Optimization

**Figure 7:**
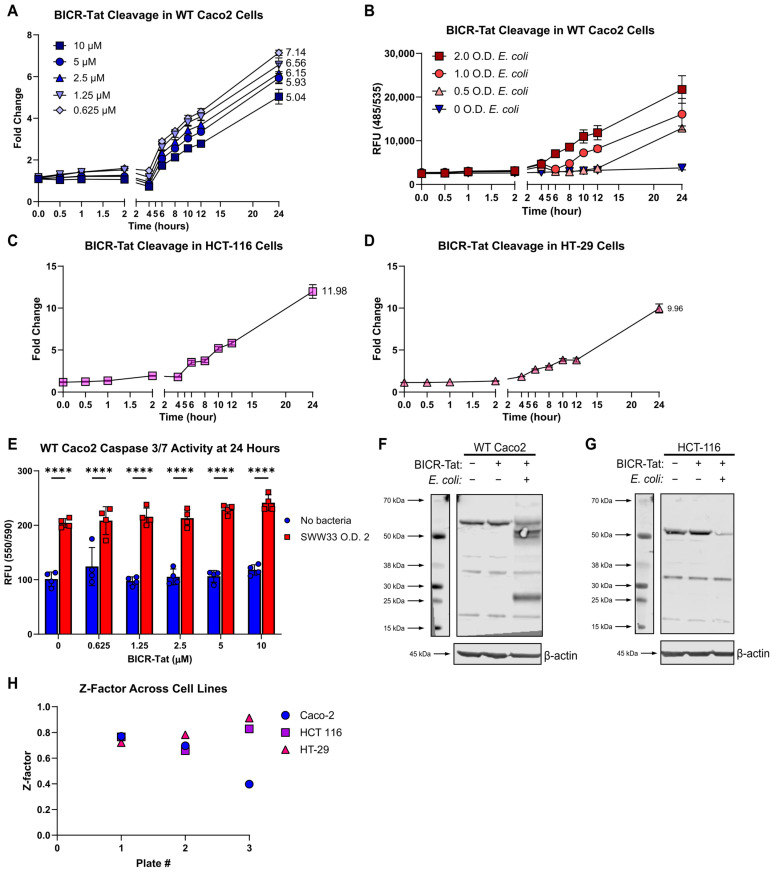
BICR-Tat cellular assay optimization. (**A**), Time course of BICR-Tat cleavage at varying concentrations of the reporter, represented as fold change over control. Fold change at 24 hpi noted on the graph for each BICR-Tat concentration. (**B**), Time course of BICR-Tat (0.5 μM) cleavage with a range of bacterial concentrations (Optical Density 600 nm; O.D.600). (**C**,**D**), Time course of BICR-Tat (0.5 μM) cleavage in HCT-116 (**C**) and HT-29 (**D**) cells. Represented as fold change over control. Fold change at 24 hpi noted on the graph. (**E**), Caspase-3/-7 activity at the 24 h endpoint of the time course (**A**). (**F**,**G**), IB of Beclin-1 cleavage products at 4 hpi in WT Caco2 (**F**) and HCT-116 (**G**) cells transfected with His-tagged Beclin-1, immunoblotted for His. (**H**), Z-factor scoring across 3 independent experiments from (**B**,**C**,**D**) with a BICR-Tat concentration of 0.5 μM and bacterial concentration (O.D.600) of 2.0. Results reported as the mean ± SD and representative of 3 independent experiments with *n* = 4. **** *p* < 0.0001 according to a two-way ANOVA.

The purpose of this study was to develop an assay capable of screening thousands of drug candidates in a high-throughput format. This necessitated optimizing performance characteristics, ensuring assay robustness, and minimizing cost per well. We started by selecting black-walled clear-bottom tissue culture plates that offered acceptable performance at a much lower cost than the optical-bottom plates used initially. Next, we tested a range of BICR-Tat concentrations to determine the lowest concentration offering acceptable signal-to-noise characteristics. All concentrations tested offered good signal-to-noise characteristics and suggested that a concentration of 0.5 µM would be sufficient for detection (Figure 7A). We verified that 0.5 µM of BICR-Tat was sufficient for detection and evaluated a range of bacterial concentrations in the assay (Figure 7B). BICR-Tat cleavage was dependent on the number of bacteria in the assay, with the highest signal generated by the largest number of bacteria. The O.D. 2.0 was chosen as the optimized concentration due to the fact that it provided excellent signal-to-noise and was linear over the signal range. This set of experiments also confirmed that 0.5 µM of BICR-Tat provided sufficient dynamic range to detect both decreased and increased BICR-Tat signals. This was a much lower amount of reagent with a large reduction in assay cost on a per-well basis. To this point, the BICR-Tat assay had proven highly robust over many different Caco2 passages, bacterial cultures, and independent plate runs. To ensure that it also performed consistently across other cell lines, we performed the optimized assay in the HCT-116 and HT-29 cell lines (Figure 7C,D). Results were consistent with those obtained using Caco2 cells in both instances.

We expected that BICR-Tat cleavage is indicative of Beclin-1 cleavage and cell death. Our finding that BICR-Tat cleavage correlated with cell counts in our assay supported this assumption. However, we wanted to confirm that BICR-Tat cleavage is associated with activation of PCD mechanisms. Measurement of executioner caspase activity at the assay endpoint demonstrated that PCD was activated in our assay under the same conditions as BICR-Tat cleavage and that the BICR-Tat itself did not influence Caspase-3/-7 activity (Figure 7E). We also investigated endogenous Beclin-1 cleavage in the presence of BICR-Tat. The kinetics of the BICR-Tat assay suggested that reporter cleavage did not start until about 4 hpi.

Immunoblotting at 4 hpi for native Beclin-1 had demonstrated loss of protein, but we were unable to detect Beclin-1 cleavage fragments and confirm protein degradation. The most likely explanations were that our antibody was unable to detect Beclin-1 fragments or that Beclin-1 was completely degraded at that point in time. This led us to transfect cells with a His-tagged Beclin-1 construct and immunoblot for His to look for protein fragmentation. In most experiments with both Caco2 and HCT-116 cells, we saw that the transfected protein was completely degraded at 4 hpi, but at 3 hpi, it was completely intact (Figure 7F,G). We were able to detect Beclin-1 fragments in one assay in Caco2 cells at 4 hpi. This suggested to us that native Beclin-1 is fully degraded quickly after the initial calpain cleavage event in this model, that cleavage is almost always complete by 4 hpi, and that BICR-Tat cleavage follows the full degradation of endogenous full-length Beclin-1. This is ideal since it means that BICR-Tat cleavage is truly indicative of the switch between Beclin-1 functional states. Finally, we evaluated BICR-Tat assay performance in the three different cell lines with a bacterial O.D. 2.0 and BICR-Tat concentration of 0.5 µM. All of the assay plates had a Z-factor of >0.4, and eight of the nine plates had a Z-factor of >0.5, indicating an excellent assay (Figure 7H). This is particularly encouraging since this assay uses both live mammalian cells and live bacteria, and each plate was prepared completely independently. Thus, the BICR-Tat assay has the characteristics needed for high-throughput screening.

### 3.8. BICR-Tat Assessement in an IBD Therapeutic Context

Our ultimate goal is to identify therapeutic candidates that protect IECs and promote intestinal barrier repair in IBD. This kind of approach is important because intestinal barrier restoration is vital for tissue healing, and very few therapeutics target this aspect of the disease. To our knowledge, no current therapies target PCD mechanisms triggered by Beclin-1 cleavage. This made it impossible to test a current IBD therapy with the desired activity in our assay. Instead, we identified a failed IBD therapy to test in our assay. This was feasible because the dynamic range of the BICR-Tat assay makes it possible to identify candidates that decrease or increase Beclin-1 cleavage. Nuclear factor kappa B (NF-kB) inhibitors were developed for use in IBD after IBD-associated genetic polymorphisms were identified in upstream signaling pathways, and high levels of NF-kB-regulated cytokines were reported in IBD patients [44]. However, it became clear that direct, indiscriminate NF-kB inhibition exacerbates disease by compromising IEC survival mechanisms and contributing to intestinal barrier failure [45].

**Figure 8:**
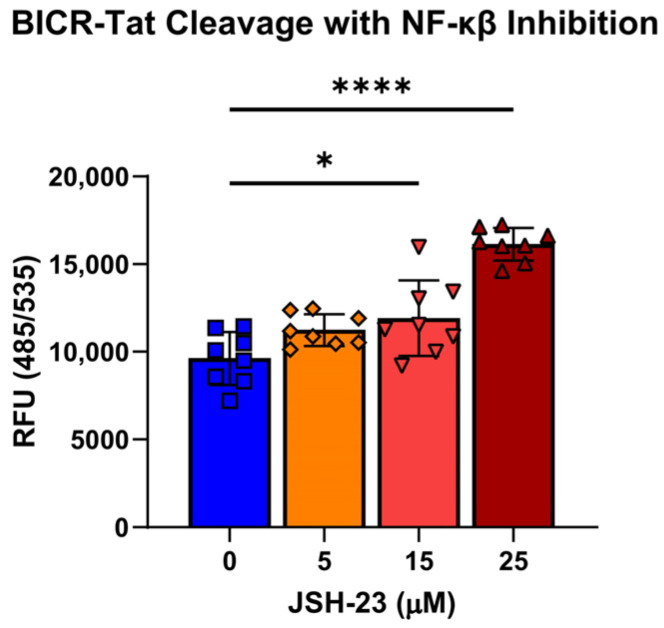
BICR-Tat assessement in an IBD therapeutic context. Fluorescent signal from BICR-Tat cleavage in infected WT Caco2 cells treated with NF-kB inhibitor and JSH-23 at the indicated concentrations. Cells were assayed at 24 hpi. Results reported as the mean ± SD and representative of 3 independent experiments performed with *n* = 8. * *p* < 0.05 and **** *p* < 0.0001 according to a one-way ANOVA.

Our model of cell stress is presumed to activate NF-kB through MAMP-triggered pattern recognition receptor (PRR) signaling. We decided to test whether our assay system could uncover the risk of NF-kB inhibition by JSH-23, an inhibitor of NF-kB (p65 subunit) translocation to the nucleus during microbial stress. In the presence of JSH-23, BICR-Tat cleavage was increased over cleavage by bacteria alone, indicating that NF-kB inhibition increased IEC death during microbial stress (Figure 8). This means that the BICR-Tat assay has the potential to identify drug candidates that have the potential to improve or exacerbate intestinal barrier integrity in IBD. These candidates could then be further evaluated in well-established, lower throughput assays of intestinal barrier function.

## 4. Discussion

In this study, we discovered that exposure to live commensal bacteria triggers calpain-mediated Beclin-1 cleavage with autophagy failure in IEC. Calpains are triggered directly and indirectly when cells are exposed to live bacteria. Bacterial MAMPs trigger calpain activation when detected by PRR, such as toll-like receptor (TLR) 2 [20]. Signaling through TLR2 generates a calpain-activating calcium flux via the release of intracellular calcium stores. Damage to cell membranes caused by bacterial porins or invasion of whole bacteria into cells allows calcium entry from the extracellular environment and activates calpains [23,46]. Bacteria may also inject effector proteins into host cells to facilitate their entry [47]. These virulence factors can decrease levels of the endogenous calpain inhibitor calpastatin, and/or trigger calpain-activating calcium changes in the cytosol. Finally, bacteria can activate inflammatory caspases that cause pores to form in the cell membrane. These pores then allow for calcium entry into cells and trigger calpain activation [48]. In our model, we did not map specific pathways of calpain activation, but we presume that several calpain-activating mechanisms are triggered simultaneously by the live bacteria. It is also likely that the number of bacteria and amount of calpain stimulation increased over the time period of our experiments since the *E. coli* doubling time is approximately 90 min. For these reasons, we suspect that calpain activity overwhelmed the inhibition provided by a single calpain inhibitor over the 24 h that our IECs were exposed to bacteria.

Activated calpains play complex roles in both cell survival and death pathways in infected IECs. Calpains are necessary for NF-kB activation, which leads to pro-inflammatory and pro-survival programs in IECs [49]. Conversely, calpains can also trigger PCD pathways when levels of active calpain overcome endogenous inhibitors and escape regulation [48]. Programmed cell death is vital to containing infection since it denies bacteria the resources that they need to replicate and can trap them within the remains of dead cells to facilitate clearance [50]. One way in which calpains contribute to PCD is through cleavage of the autophagy proteins Beclin-1 and Atg5. Autophagy, or more specifically, xenophagy, envelops intracellular bacteria in a double membrane vesicle for destruction [51]. Autophagy is also responsible for the removal of pro-inflammatory signaling complexes, such as inflammasomes, generated in cells responding to infection [52]. When either of these autophagic processes fails, IECs die due to overwhelming damage or inflammation. Beclin-1 and Atg5 cleavage also generate protein fragments that directly trigger PCD [53,54]. Intracellular, cytosolic HMGB1 can limit calpain-mediated inactivation of autophagy [15]. However, this protection can fail when HMGB1 is actively exported from cells in response to PRR signaling or when levels of active calpain exceed the regulatory capacity of HMGB1 in the cell cytosol [55]. In this study, levels of intracellular HMGB1 were similar in unchallenged and infected cells at 4 hpi, when full-length Beclin-1 and p62 were already depleted in infected IEC. This suggests that both calpains and caspases were already activated at this timepoint and likely contributing to PCD in our model. It is possible that levels of active calpain overwhelmed HMGB1 protection or that multiple PCD pathways were simultaneously activated and HMGB1 regulation of Beclin-1 and Atg5 cleavage was not sufficient to rescue IECs from PCD under these conditions.

We have demonstrated that BICR-Tat, a cell-penetrant nanosensor derived from the calpain-cleaved region of Beclin-1, reports the switch from autophagy to apoptosis in microbe-stressed IECs in real time and after fixation. Our ultimate goal is to use BICR to identify therapeutics with the potential to promote intestinal barrier restoration in IBD. Current IBD therapies are primarily directed at suppression of immune activity within the intestine [56,57,58]. While these therapies are often able to achieve clinical remission, sustained remission with complete intestinal healing or cures have not been achievable with these regimens. These drugs are also often associated with side effects due to immunosuppression. Thus, novel therapies targeting IECs and designed to promote intestinal barrier restoration without immunosuppression would be a major advancement and useful addition to IBD therapeutic regimens. Previous studies of IECs within IBD lesions have demonstrated that Beclin-1 cleavage is a specific mechanism of PCD that is highly relevant to this disease. While targeting calpains directly would be the most straightforward approach to improving IEC survival during microbial stress, this approach has several drawbacks. Most notably, calpains are required for epithelial repair. Additionally, inhibiting ubiquitous calpains may not be sufficient since intestinal epithelial cells produce seven different calpain family members, Calpain-1, -2, -5, -7, -9, -13, and -15 [59]. It is also possible that calpain activation occurs within a complex network of interconnected proteases that must be addressed to improve IEC survival. For these reasons, we believe that focusing on preserving key calpain substrates, like Beclin-1, is the preferred approach to therapeutic development.

While BICR performed well in our study and has the potential to be of great use in the search for therapeutics that promote intestinal barrier restoration, some key questions remain. First, we saw no benefit of HMGB1 in this model. We believe that this is because the number of bacteria overwhelmed the ability of HMGB1 to protect Beclin-1 from cleavage. We plan to address this in future studies by testing a range of bacterial concentrations in both HMGB1-sufficient and HMGB1-deficient cells over time. Second, calpain inhibition alone was not sufficient to decrease BICR-Tat cleavage in our model. This could be due to overwhelming calpain activity that was not inhibitable using a single calpain inhibitor, BICR cleavage by proteases other than calpain, or bacterial proteases acting on BICR that cannot be inhibited with calpain inhibitors designed for use in host cells. Finally, we utilized commensal *E. coli* in our studies, but the gut bacterial community can contain thousands of different species, and the composition varies widely across individuals. Live bacteria trigger many different pattern recognition receptors and can cause direct damage to cells. Some bacteria also manipulate calpains and autophagy for their own gain. This means that we may need to rescue Beclin-1 within the context specific to an individual patient. Fortunately, BICR is adaptable to any cell type and agnostic as to the specific calpain active in a given cell, as long as it cleaves Beclin-1. This tool can detect any cause of Beclin-1 cleavage: altered protease activity, changes in intracellular HMGB1 concentration, or HMGB1 genetic polymorphisms that affect its function. Ultimately, BICR could be a powerful tool to identify therapeutic leads for intestinal barrier repair. This has the potential to open a path to precision medicine for IBD and other diseases in which the autophagy/apoptosis checkpoint is malfunctioning and contributing to disease.

## 5. Summary and Conclusions

The goal of this study was to create an assay capable of detecting Beclin-1 cleavage in a high-throughput format. We successfully produced a live cell assay that detects Beclin-1 cleavage triggered by live bacterial stress in real time with high sensitivity and broad dynamic range. Our initial assessment suggests that this assay will perform well in high-throughput screens of compound libraries and identify hits that protect IECs from microbe-triggered PCD. Intestinal epithelial barrier integrity is the most important predictor of disease outcome in IBD. This has made barrier restoration an important therapeutic goal in the treatment of this disease. However, current therapies are not sufficient to meet this goal since most are designed to suppress inflammation rather than promote epithelial repair. Thus, the approach and tool developed in this study have the potential to open new therapeutic paths to intestinal repair that could achieve sustained remission and better quality of life for patients.

## Data Availability

The original contributions presented in this study are included in the article. Further inquiries can be directed to the corresponding author.
